# Predictability of Sequential Distalization of the Upper Arch Using Invisalign^®^: A 3D Superimposition Study

**DOI:** 10.3390/dj14090601

**Published:** 2026-09-16

**Authors:** Cristina Menéndez López-Mateos, Mª Luisa Menéndez López-Mateos, José Antonio Alarcón, Mario Menéndez-Núñez, Conchita Martin

**Affiliations:** 1 Department of Stomatology, Faculty of Odontology, University of Granada, Campus Universitario de Cartuja, s/n, 18071 Granada, Spain; menendezlmc@correo.ugr.es (C.M.L.-M.); menendez@correo.ugr.es (M.L.M.L.-M.); menendez@ugr.es (M.M.-N.); 2 BIOCRAN (Craniofacial Biology: Orthodontics and Dentofacial Orthopedics) Research Group, Complutense University of Madrid, 28040 Madrid, Spain; mariacom@ucm.es; 3 Section of Orthodontics, Department of Dental Clinical Specialties, Faculty of Odontology, Complutense University of Madrid, 28040 Madrid, Spain

**Keywords:** predictability, clear aligners, sequential distalization, tooth movements, 3D superimposition, virtual planning, upper arch

## Abstract

**Background**: Sequential distalization of the upper arch with clear aligners is widely used to correct Class II malocclusion, but its predictability across molars, canines, and incisors is unclear. This study compared virtual planning with intraoral outcomes and identified the associated side effects. **Methods**: A retrospective study analyzed 80 maxillary quadrants from 40 patients with dental Class II malocclusion treated with Invisalign^®^ (Smart Track^®^ material) using a sequential V-pattern distalization protocol with Class II elastics and vertical rectangular/optimized attachments. Predicted movements were extracted from ClinCheck Pro^®^. Scans at baseline and after the first approved aligner stage were superimposed on the palatal rugae using Geomagic Control X^®^. Extrusion/intrusion, buccolingual movement, mesiodistal translation, rotation, angulation, and torque were measured for molars, canines, and incisors. **Results**: Molar distalization reached 1.80 of the 2.60 mm planned (69.2%), and canines 1.12 of 2.45 mm (45.7%) and incisor intrusion reached only 0.03 of the 1.45 mm planned (2.1%). Buccolingual molar movement was most predictable (97.4%), and molar rotation showed moderate-to-good predictability (88.4%); incisor rotation, angulation and torque accuracy were 62.2%, 53.8% and 23.2%, respectively. Molar angulation and torque were markedly overexpressed, with unplanned intrusion of 0.80 mm; canine angulation/torque were similarly overexpressed. Incisor intrusion/torque movements were least predictable. **Conclusions**: Predictability of sequential upper-arch distalization varied widely across tooth groups and movement types. Linear distalization was moderately to poorly predictable and rotational movements were well reproduced, but angulation/torque control were the least predictable, especially for molars and canines, with unintended molar intrusion and buccal torque. Incisor torque, intrusion, and retrusion were most underexpressed. Vertical rectangular attachments improved molar tipping control, while absent attachments and Power Ridges worsened incisor torque/translation. Clinicians should anticipate overcorrection and refinement stages to avoid false expectations based on the initial ClinCheck outcome.

## 1. Introduction

Molar distalization remains one of the most challenging tasks in orthodontics [1]. Achieving bodily molar movement requires that the applied force passes through the tooth’s center of resistance, or that an equivalent system of forces and moments is accurately applied to the crown of the tooth [2]. Clear aligner therapy (CAT) has been suggested as an effective tool for molar distalization, probably due to its full coverage of the dental crown, which enhances control of movement. Rossini et al. [3] reported that distalization with CAT is generally predictable, while Simon et al. [4] and Saif et al. [5] reported an accuracy of 76–87% using digital model analysis.

Ravera et al. [6] concluded that, when combined with Class II elastics and attachments, upper first molars can be distalized about 2.25 mm without significant tipping or vertical crown displacement, as evaluated on lateral cephalograms. Similarly, Caruso et al. [7] observed stable vertical control while achieving mass molar distalization of about 3 mm.

Most previous investigations evaluating clear-aligner distalization present three main limitations: they have focused primarily on the molar segment in isolation without assessing sequential effects on canines and incisors; outcomes were typically measured immediately after the distalization phase, without accounting for vertical and angular side effects such as crown tipping, intrusion, and torque changes that accompany anchorage loss; and few studies have evaluated these movements within the context of a complete Class II treatment, limiting the clinical applicability of their findings to real-world sequential protocols [8,9,10,11].

The objective of this study was to determine whether the tooth movements predicted by virtual planning—including, but not limited to, molar distalization—were accurately reproduced intraorally over the full course of the sequential distalization protocol, evaluating molars, canines, and incisors together.

## 2. Materials and Methods

To address the gaps identified above, this study employed advanced 3D digital superimposition techniques to assess treatment outcomes throughout the upper arch, evaluating molars, canines, and incisors together—including their three-dimensional linear and angular displacements and their interrelated biomechanical effects—within the context of a complete sequential V-pattern distalization protocol.

### 2.1. Study Design and Sample

A longitudinal retrospective study was performed on a consecutive series of patients treated with clear aligners. Patients were consecutively recruited in 2025. All diagnoses and treatment plans were carried out by a single orthodontist (MMN), using a sequential distalization protocol.

A sample of 40 patients (80 hemiarches) meeting the inclusion criteria described below was included. The sequential distalization protocol followed a stepwise, V-pattern staging: distalization begins with the second molar; once approximately one-third to one-half of its planned movement has been achieved, the first molar is engaged; this is followed sequentially by the premolars, the canine, and finally the incisors without overcorrection. The prescribed sequential V-pattern distalization protocol targeted a mean distal movement of 2.60 mm for the first molars and 2.45 mm for the canines (Table 1 and Table 2). A mean buccolingual movement of 1.17 mm was also prescribed for the first molars, reflecting the transverse expansion. The mean number of aligners used was 46.

### 2.2. Inclusion and Exclusion Criteria

Patients were selected based on the following inclusion criteria: adults with complete permanent dentition, bilateral dental Class II malocclusion, treatment with Invisalign^®^ clear aligners made of Smart Track^®^ (Align Technology, Santa Clara, CA, USA) material using the sequential V-pattern distalization protocol, and either absence or prior extraction of upper third molars.

Exclusion criteria included: previous orthodontic treatment, tooth extractions other than third molars, presence of implants or fixed prostheses in the upper arch, or craniofacial deformities/syndromes.

### 2.3. Sample Size Calculation

A power analysis was performed considering the efficacy of maxillary molar distalization as the primary outcome. Based on the results for the mean and standard deviation reported by Miao et al. [12], and assuming an alpha of 0.05 and 80% power, a total sample size of 80 molars was required. Since measurements were bilateral, 40 patients (80 molars) provided the necessary sample size.

### 2.4. Sequential V-Pattern Distalization Protocol

Sequential V-pattern distalization was prescribed in the upper arch to correct Class II malocclusion. A V-pattern was used to achieve two-thirds molar distalization without overcorrection. Patients were instructed to wear clear aligners for at least 22 h/day, changing them every 10 days [6]. Class II elastics (3/16-inch, medium force, 4.5 oz) for 22 h/day were prescribed from precision cuts on the upper canines to bonded buttons on the lower first molars. Elastic wear was initiated at the start of upper first molar distalization and continued through the anterior retrusion phase.

### 2.5. Digital Records and Measurements

Intraoral scans were obtained at two time points: baseline (T0) and after the completion of the first prescribed aligner set, corresponding to the planned sequential V-pattern distalization of the upper arch and prior to any refinement (T1), using the iTero™ Element 2 scanner (Align Technology, San Jose, CA, USA).

3D intraoral models were exported in STL format with HD resolution using OrthoCAD 3.5 software (Align Technology, Santa Clara, CA, USA). Measurements were performed using Geomagic Control X^®^ (Geomagic Qualify 2019, 3D Systems, Rock Hill, SC, USA), a validated software for 3D measurements [13].

### 2.6. Superimposition Protocol

**Figure 1:**
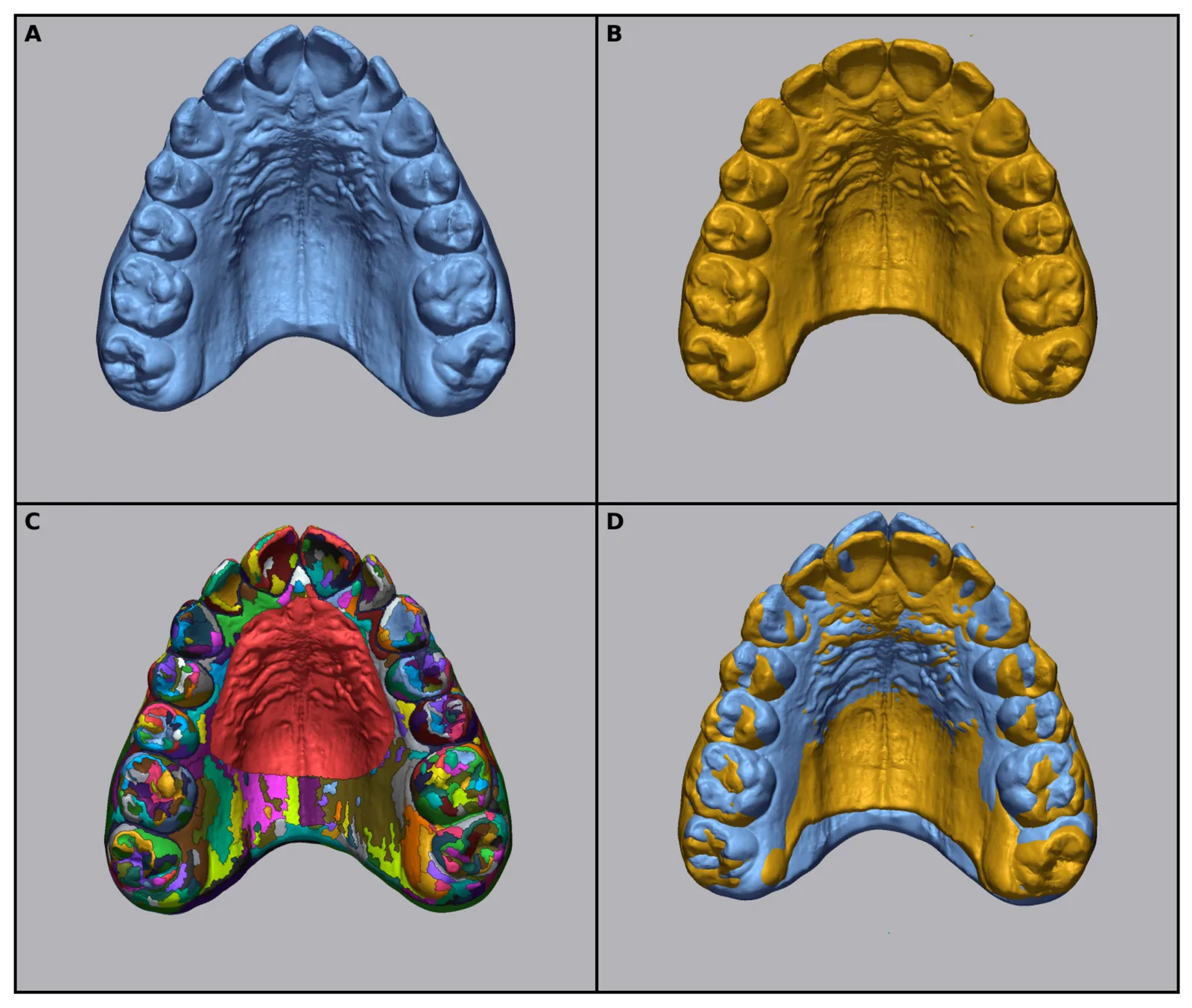
The superimposition of the pretreatment model and the model after distalization. (**A**) Pretreatment. (**B**) Second scan after maxillary molar distalization. (**C**) Selecting the palatal rugae area. (**D**) Local best fit after selecting the palatal rugae area of the two models. The colors represent the different segmented surface patches automatically generated during best-fit area selection, with the palatal rugae reference area highlighted in red.

Initial and final stage models were first pre-aligned [14]. Final superimposition was performed using the local best-fit function after selecting the palatal rugae region as the reference area [15,16] (Figure 1). After aligning both images (T0 and T1), a coordinate system was created to map the movements over three dimensions. This system was designed to match the coordinate system of the ClinCheck Pro^®^ tooth movement table (Align Technology, Santa Clara, CA, USA). In this system, the X-axis represents the buccolingual direction, the Y-axis represents the mesiodistal direction, and the Z-axis represents the occlusogingival direction.

**Figure 2:**
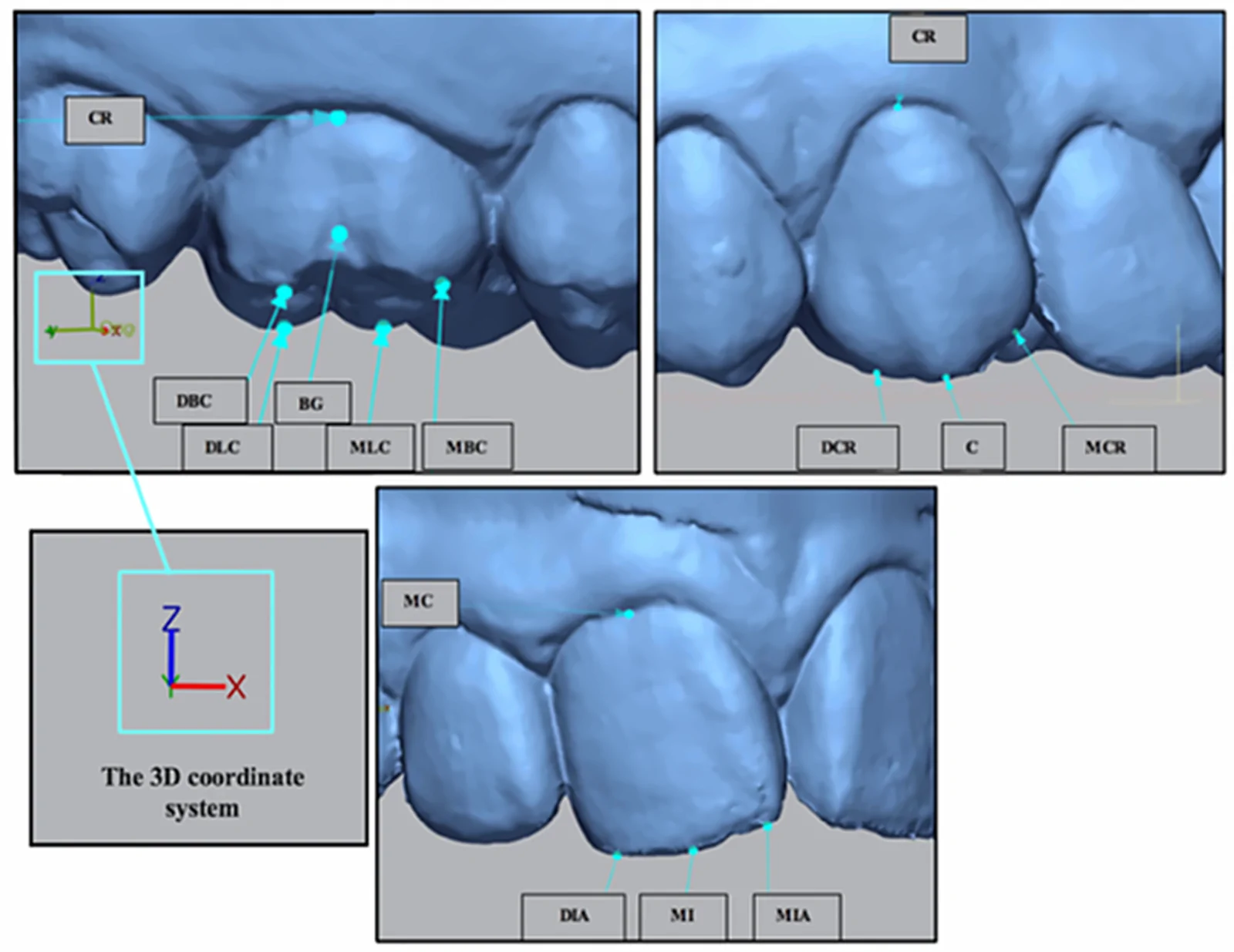
MBC, Mesiobuccal Cusp of Upper Molar; DBC, Distobuccal Cusp upper first molar; MLC, Mesiolingual Cusp upper first molar; DLC, Distolingual Cusp upper first molar; BG, Buccal groove upper first molar; CR, Cervical Ridge upper first molar; MCR, Mesial Cups Ridge Upper Canine; DCR, Distal Cups Ridge Upper Canine; C, Cusp Upper canine; CR, Cervical Ridge Upper Canine; MIA, Mesial incisal Angle Upper Incisor; DIA, Distal Incisal Upper Incisor; MI, Mid Incisal Upper Incisor; MC, Mid Cervical Upper Incisor.

A total of 14 digital landmarks per hemiarch were identified (Figure 2): six on molars, four on canines, and four on central incisors. Thus, 2240 points were analyzed (40 patients, 80 hemiarches).

#### 2.6.1. Linear Measurements

**Figure 3:**
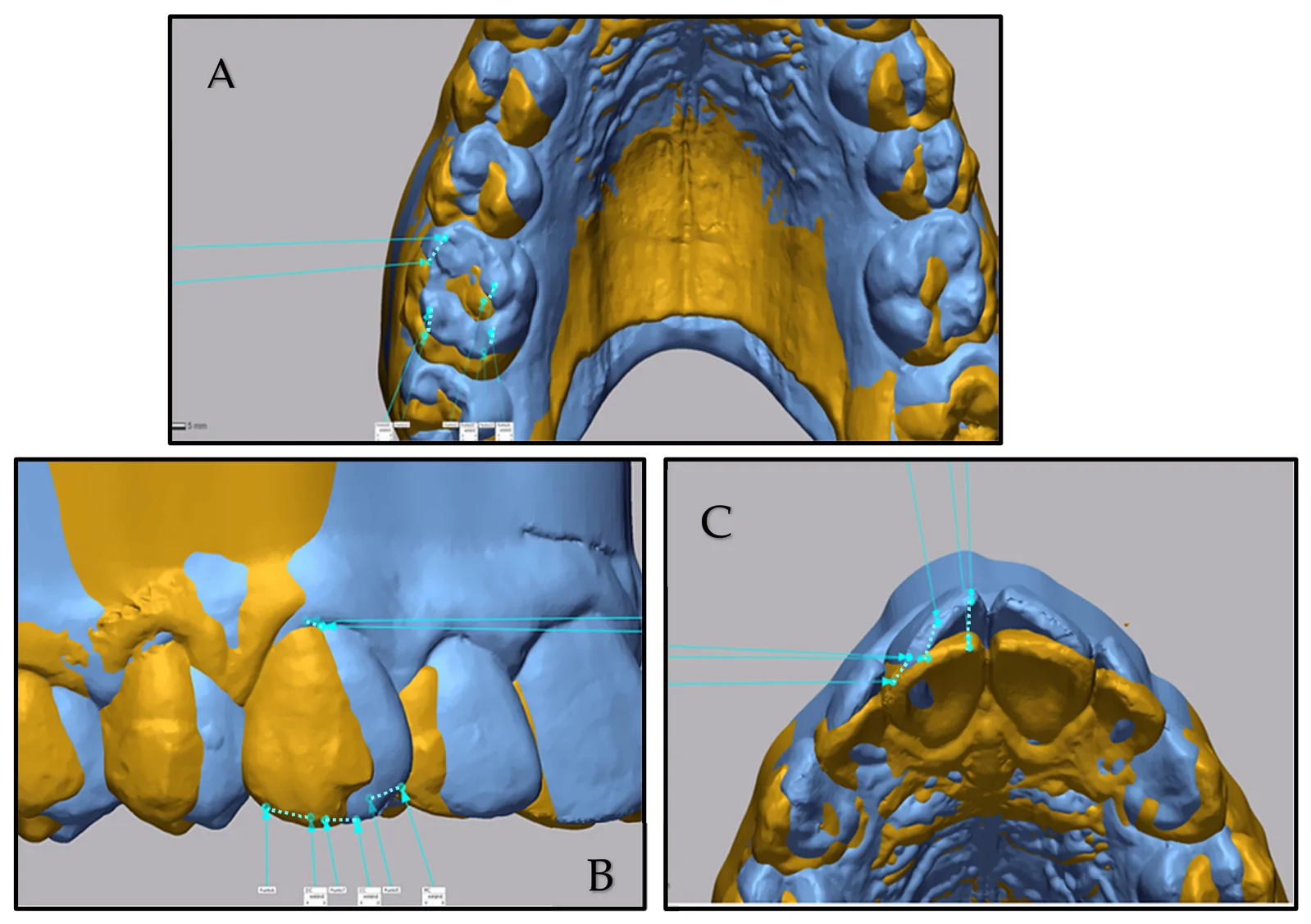
Caption. Representative 3D superimpositions used for linear measurements. (**A**) Mesial/distal translation, Bucco/lingual translation, Extrusion/intrusion of the molar. (**B**) Mesial/distal translation, Bucco/lingual translation, Extrusion/intrusion of the canine. (**C**) Mesial/distal translation, Bucco/lingual translation, Extrusion/intrusion of the incisor. Blue and gold/orange surfaces represent the T0 (pretreatment) and T1 (post-distalization) scans, respectively.

Tooth displacement was calculated as the distance between T0 and T1 reference points (Figure 3). Displacements were projected on the X-axis (buccolingual translation), Y-axis (anteroposterior translation), and Z-axis (vertical movement: extrusion/intrusion). Movements were considered positive if they corresponded to distalization, intrusion or buccal displacements.

#### 2.6.2. Angular Measurements

**Figure 4:**
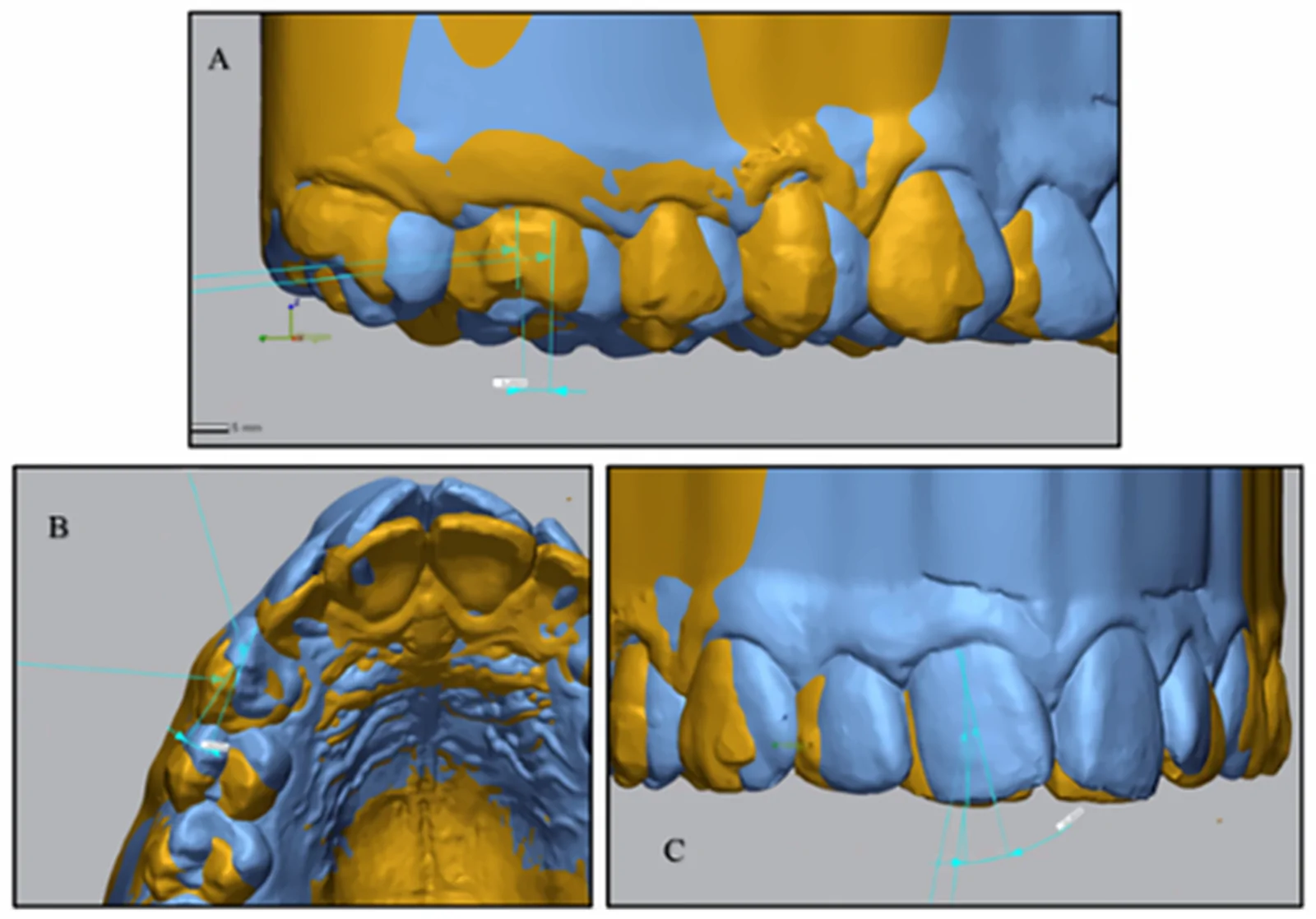
Representative 3D superimpositions used for angular measurements. (**A**) Molar rotation, angulation, and torque. (**B**) Canine rotation, angulation, and torque. (**C**) Incisor rotation, angulation, and torque.

Angular changes were quantified in Geomagic following the methodology of Lucchese et al. [17]. The angulation was measured as the angle between tooth axes at T0 and T1 projected onto three planes: Y–Z (mesiodistal angulation), X–Y (rotation), and X–Z (buccolingual torque). Figure 4 illustrates the methodology applied (Figure 4).

### 2.7. Statistical Analysis

To assess intra-examiner reliability, 20% of the digital models were randomly selected and remeasured by the same examiner after a one-week interval. Reliability was assessed using the intraclass correlation coefficient (ICC).

Quantitative data were summarized using means, standard deviations, and 95% confidence intervals; categorical data were described with frequency distributions. The Kolmogorov–Smirnov test was used to verify normality. Categorical variables were compared with chi-square tests.

Normally distributed linear and angular tooth-movement variables were compared between predicted and achieved values using the paired Student’s *t*-test, whereas non-normally distributed variables were compared using the Wilcoxon signed-rank test. A multivariate linear mixed model was performed to evaluate the influence of predictors (ANB, mandibular plane, number of aligners, overbite, overjet, presence of attachments/Power Ridges) as fixed effects on actual tooth movement, with predicted movement defined as a random effect. The ANB angle and mandibular plane angle were included as explanatory variables because they represent the sagittal and vertical skeletal patterns of the patients, respectively. These factors may influence the biomechanical response to sequential distalization and were therefore considered potential predictors of tooth movement predictability. All analyses were conducted using SPSS version 30.0 (IBM Corp., Chicago, IL, USA). Statistical significance was set at *p* < 0.05.

## 3. Results

The sample comprised 40 patients (31 women, 9 men; 80 hemiarches). The mean age was 33.21 years, with a mean ANB angle of 4.97° (reflecting a mild skeletal Class II pattern) and a mean mandibular plane angle of 30.26°. The mean overjet was 3.77 ± 1.49 mm and the mean overbite was 3.80 ± 2.32 mm. The mean number of aligners used was 46.

The intrarater reliability was excellent, with an ICC value of 0.908 (*p* < 0.001).

### 3.1. Molar Movements

Table 1 summarizes the predicted, achieved, and deviational movements of the upper first molars. A mean intrusion of 0.80 mm was observed, whereas an extrusion of 0.01 mm had been predicted (*p* < 0.001). Buccolingual translation was close to the prediction (1.14 mm vs. 1.17 mm; *p* = 0.462). Distal translation was significantly lower than planned (1.80 mm achieved vs. 2.60 mm predicted; *p* < 0.001). Mesio-buccal rotation was 5.55° compared to the 6.28° planned (*p* < 0.001). Distal angulation was greater than expected (6.51° vs. 2.83°; *p* < 0.001). Finally, buccal crown torque was significantly higher than predicted (4.12° vs. 0.57°; *p* < 0.001).

### 3.2. Canine Movements

The results for canines are shown in Table 2. A slight extrusion of 0.03 mm occurred, compared to a planned intrusion of 0.12 mm, with no significant difference (*p* = 0.337). Buccolingual translation showed no net change, despite a planned 0.35 mm lingual displacement (*p* < 0.001). Distal translation was significantly lower than predicted (1.12 mm vs. 2.45 mm; *p* < 0.001). Mesio-buccal rotation was reduced (5.14° vs. 9.61°; *p* < 0.001). Distal tipping exceeded the planned value (2.80° vs. 0.69°; *p* < 0.001). Finally, buccal crown torque was slightly greater than predicted (4.20° vs. 3.20°; *p* < 0.001).

### 3.3. Incisor Movements

Table 3 shows the predicted and achieved incisor movements. Actual intrusion was minimal (0.03 mm), while 1.45 mm had been planned, resulting in a significant deviation (*p* < 0.001). Buccolingual translation was in the opposite direction of the prediction (0.09 mm buccal vs. 0.76 mm planned lingual; *p* < 0.001). Distal translation was 0.07 mm compared to a planned 0.13 mm, without significant difference (*p* = 0.628). Rotation (1.63° vs. 2.62°; *p*= 0.184) and distal angulation (0.91° vs. 1.69°; *p* < 0.001) showed limited predictability. Torque expression was substantially lower than predicted (1.76° vs. 7.59°; *p* < 0.001).

### 3.4. Predictability

Table 4 summarizes the predictability of each assessed movement, expressed as the percentage of the planned movement that was actually achieved.

Among the linear movements, buccolingual translation of the molars showed the highest predictability (97.4%), whereas mesiodistal translation (distalization) was moderately predictable for molars (69.2%) and considerably less predictable for canines (45.7%). Incisor intrusion showed very low predictability (2.1%), with the planned movement largely unexpressed. For angular movements, molar and canine rotation were reasonably predictable (88.4% and 53.5%, respectively), while angulation (mesiodistal tipping) and torque were markedly overexpressed relative to the plan in both molars and canines. In contrast, incisor torque was substantially underexpressed (23.2%). For molar extrusion/intrusion, canine extrusion/intrusion and buccolingual translation, and incisor buccolingual translation, the achieved movement occurred in the opposite direction to that which was planned. For canine extrusion/intrusion and incisor mesiodistal translation, the planned value was close to zero, precluding a clinically meaningful ratio.

### 3.5. Multivariable Analysis

Results of the multivariable linear regression models evaluating potential factors influencing tooth movements are presented in Table 5, Table 6 and Table 7.

Molars (Table 5). Most of the evaluated variables were not significantly associated with molar movement outcomes. However, the mandibular plane angle showed a significant negative association with molar rotation (B = −0.212; 95% CI: −0.372 to −0.052; *p* = 0.010), indicating that patients with higher mandibular plane angles tended to exhibit less molar rotational movement. In addition, attachment design significantly influenced molar angulation. Compared with vertical rectangular attachments, optimized attachments were associated with a significant increase in molar inclination, indicating reduced control over this movement (B = 1.430; 95% CI: 0.031 to 2.828; *p* = 0.045). No significant associations were found for ANB angle or number of aligners.

Canines (Table 6). Overbite showed a significant negative association with canine angulation (B = −0.846; 95% CI: −1.333 to −0.359; *p* = 0.001) and a marginal association with mesiodistal translation (B = −0.071; 95% CI: −0.147 to 0.003; *p* = 0.062). Overjet was also significantly associated with canine angulation (B = −1.202; 95% CI: −2.024 to −0.380; *p* = 0.005). No significant effects were identified for attachment type or ANB angle.

Incisors (Table 7). Overbite was significantly associated with both mesiodistal translation (B = −0.088; 95% CI: −0.158 to −0.018; *p* = 0.014) and extrusion/intrusion (B = 0.138; 95% CI: 0.040 to 0.236; *p* = 0.014). Overjet also significantly influenced extrusion/intrusion (B = −0.189; 95% CI: −0.310 to −0.068; *p* = 0.003). In addition, the absence of incisor attachments was associated with a significant reduction in torque expression (B = −2.422; 95% CI: −4.232 to −0.612; *p* = 0.009), while the absence of Power Ridges had an even greater negative effect on torque control (B = −3.788; 95% CI: −5.636 to −1.940; *p* < 0.001). No significant associations were found between ANB angle and any of the evaluated incisor movements, nor between attachment variables and translational or rotational movements.

## 4. Discussion

The present study aimed to assess the predictability of the sequential V-pattern distalization in the upper arch, including first molars, canines and incisors, and to investigate the influence of clinical and biomechanical factors such as type of attachment (optimized or rectangular), Power Ridges, ANB angle, mandibular plane angle, and the number of aligners. In this way, we aimed to provide a more comprehensive perspective on the sequential distalization process, considering the entire treatment sequence and including molars, canines, and incisors. These discrepancies highlight the complexity of achieving sequential distalization in a full Class II treatment context.

In the present sample, maxillary first molars were distalized 1.80 mm compared to the 2.60 mm planned (69.2% accuracy). This is lower than the 88.4% accuracy reported by Simon et al. [4], who found maxillary molar distalization to be most effective when more than 1.5 mm was prescribed, but consistent with D’Antò et al. [18], who found accuracies of 69.4% and 75.2% for first and second molars, respectively. Ravera et al. [6], using cephalometric overlays, reported effective distalization (2.25 mm for the first molars and 2.52 mm for the second molars), although their methodology differed from ours.

The accuracy of molar distalization in this study was lower than that reported in previous investigations. Differences between studies may relate to observation periods and to the loss of posterior anchorage inherent to sequential distalization, since virtual planning (ClinCheck) assumes idealized conditions, whereas in vivo movement is influenced by biomechanical side effects. Although our study did not directly quantify anchorage loss, Li et al. [19] reported that retraction mechanics markedly reduced molar distalization accuracy (31.5% versus 48.1% without retraction). While their protocol differs from ours and direct comparisons should be made with caution, our findings are consistent with previous reports suggesting that anchorage demand may influence distalization predictability.

### 4.1. Biomechanical Interpretation of Molar Movements

Distal tipping, intrusion, and buccal torque were consistent findings in molar movements. In our study, molars exhibited an additional distal tipping of 3.69°, intrusion of 0.80 mm, and buccal torque of 3.55°. Biomechanically, distal tipping induces intrusion of the distal cusp and extrusion of the mesial cusp; however, aligner coverage of the occlusal surface prevents mesial cusp extrusion, resulting in overall intrusion. These patterns corroborate finite element simulations [20] and clinical studies [12] showing that clear aligner distalization induces buccal torque, distal inclination, and intrusion.

The type of attachment also influenced molar behavior. Vertical rectangular attachments (VRAs) provided better control of molar inclination compared to optimized attachments. Previous studies support the use of VRAs for effective distalization [4,6,21], although some authors argue they may not always be necessary [22]. Attachments enhance aligner fit, particularly in short clinical crowns, improving force expression and reducing uncontrolled crown tipping. Finite element analyses [20,23] further confirm that VRAs distribute forces more effectively than optimized designs, making them the most suitable choice for molar distalization. Overall, molar distalization was less than planned, accompanied by greater crown distal inclination, intrusion, and buccal crown torque—side effects that reflect the biomechanical repercussions of distalization beyond the intended displacement alone.

The sequential distalization protocol used in our study allows for both simultaneous molar derotation and transverse expansion of the posterior teeth, thus contributing to the correction of Class II malocclusions. Predictability of mesio-buccal derotation was high (88.4%), in line with D’Antò [18], who reported an accuracy of 77.5%, and Lione et al. [24], who confirmed reliable derotation with aligners when combined with expansion and optimized attachments. The relatively high predictability of molar derotation may be related to the morphology of maxillary molars. Because the center of resistance is located closer to the rotational axis than in translational movements, lower moments are required to achieve rotational correction. Buccolingual translation was the most predictable molar movement, with an accuracy of 97.4% (1.14 mm achieved vs. 1.17 mm planned). These results are consistent with Morales-Berruezo et al. [25], who demonstrated high predictability for expansion movements, especially when the magnitude of expansion is moderate, and with recent systematic evidence showing that first molar rotations achieve the highest predictability among posterior teeth, generally exceeding 75% accuracy [26].

### 4.2. Canine Movements

During sequential distalization of the upper arch, it was observed that canine distalization was poorly expressed (1.12 mm achieved vs. 2.45 mm planned; 45.7% accuracy, with a significant difference of 1.33 mm). In addition, the canine crown presented an unexpected inclination of 2.11° towards the distal, as well as translation and buccal torque movements that were not initially planned. As the aligner sequence progressed, the predictability of the movements decreased progressively. These findings can be explained by anchorage loss from molar distalization, as reported by Miao et al. [27], who concluded that as the unachieved distal movement of the first molar increases, there is a tendency toward greater mesial movement of the premolars and canine. Similarly, Ren et al. [28] in their study with premolar extractions revealed that, compared to planned tooth movements, the canines showed less distalization, greater distal inclination, lingual inclination, mesial rotation, and extrusion. Rotational accuracy in canines was low, with a deviation of 4.5°, likely related to their rounded crown morphology. Previous studies support this observation, including Kravit et al. [29,30], who noted that rotational movement in canines is one of the most difficult to predict, with an accuracy of 32%, indicating that teeth with a rounded morphology, particularly in treatments with aligners, are more difficult to rotate effectively, and a recent systematic review confirmed that maxillary canines consistently show the lowest rotational accuracy among all tooth types, averaging between 36% and 50% [26]. The limited contact surface between aligners and rounded crowns reduces the effectiveness of rotational forces. Overall, the canine showed considerably less distal movement than planned, with greater crown distal inclination, consistent with the anchorage loss associated with molar distalization described above [26].

### 4.3. Incisor Movements

Incisor movements were the least predictable. Planned intrusion (1.45 mm) was virtually absent (0.03 mm achieved), torque expression was markedly reduced (difference of 5.83°), and retrusion was not achieved, with a slight buccal displacement observed. During the sequential distalization process, the PIR protocol is applied to the anterior sector, consisting of a sequence of movements that includes first protrusion, followed by intrusion, and finally retrusion of the incisors. The lack of torque, intrusion, and retrusion in the anterior sector resulted in the aligner being longer than the arch, which can cause a posterior open bite. This phenomenon, known as the “bowing effect,” occurs when the length of the arch is shorter than that of the aligner, causing the aligner to bend in the center and intrude on the first premolars, second premolars, and first molars. As previously mentioned, our study observed an unexpected intrusion of 0.80 mm at the level of the first molar. Distalization is often accompanied by anterior buccal movement; however, achieving an effective PIR protocol is challenging. Based on these findings, it would be recommended to overcorrect the torque and intrusion of incisors, since the plastic used in aligners has a deficit of expression in these movements. Some authors have suggested incorporating overcorrection into the final position of the aligners to try to compensate for this lack of expression [31,32]. In our interpretation of the present data, the incisors showed no net distal movement or intrusion, and torque expression remained below the planned values, an effect amplified by the absence of attachments and Power Ridges in this sample.

The results of our study indicate that the absence of attachments and power ridges further compromised incisor control, significantly reducing torque expression and translational accuracy. These auxiliaries provide additional anchorage and improve stress distribution, which is essential for achieving adequate torque. Without them, control is insufficient and treatment stability may be compromised. This finding is consistent with previous reports, such as Sanhya et al. [33], who compared incisor torque expression in a finite element model using aligners without auxiliaries, with horizontal ellipsoidal attachments, and with power ridges. Their study found that the horizontal ellipsoid attachment achieved better torque control of the central incisor than both the power-ridge and no-auxiliary models, and that aligners without any auxiliary performed the worst. The latter finding is consistent with our own observation that the absence of attachments reduced torque expression, whereas the comparative advantage of Power Ridges over attachments for torque control was not confirmed in their model.

The multivariable analysis showed that most clinical and biomechanical variables were not significant predictors, which warrants brief discussion. The ANB angle showed no effect, likely due to the limited skeletal variability of the sample and its indirect influence on aligner mechanics. The number of aligners was also non-significant, suggesting that, within the observed range (mean 46), predictability depends more on aligner sequence design than on treatment duration itself. Overbite and overjet were included for incisors and canines due to their biomechanical relevance and were significant predictors of incisor extrusion/intrusion and mesiodistal translation (overbite, *p* = 0.014) and incisor torque/canine inclination (overjet, *p* = 0.003 and *p* = 0.005); their lesser relevance to vertical interarch relationships justified their exclusion from the molar model. Other non-significant molar movements may be more strongly governed by attachment geometry and force distribution characteristics than by attachment type alone [20,23]; aligner material properties were not directly assessed in the present study.

By addressing distalization within the overall context of complete Class II treatment, this study provides a more comprehensive perspective than analyses focused on isolated tooth movements, capturing not only intended tooth displacement but also associated side effects—such as anchorage loss, crown inclination changes, and limited expression of intrusion and torque—that are inherent in orthodontic mechanics. This integrated perspective provides a more clinically applicable basis for treatment planning, which is translated into the following practical recommendations.

### 4.4. Clinical Implications

The discrepancies identified between planned and achieved tooth movements—including unplanned molar intrusion, deficits in molar and canine distalization, and deficits in incisor torque and intrusion—carry direct implications for clinicians applying sequential distalization with clear aligners. Given the limited predictability of molar and canine distalization, intermediate review appointments should be anticipated, with additional aligners prescribed if the planned movement has not been adequately expressed by the end of the initial sequence. Vertical rectangular attachments, rather than optimized attachments, should be the default choice for molars undergoing distalization, given their superior control of crown inclination; for canines, the rounded crown morphology limits rotational control regardless of attachment type, warranting closer monitoring and consideration of additional auxiliaries when derotation is a priority. Power Ridges and incisor attachments should be incorporated whenever anterior torque or intrusion is a treatment goal, with overcorrection built into the digital setup to compensate for the systematic underexpression of these movements. Clinicians should also anticipate the risk of a bowing effect arising from unplanned posterior intrusion combined with incomplete anterior intrusion/retrusion and verify that the proclination–intrusion–retrusion (PIR) sequence is correctly programmed. More broadly, orthodontists should not assume that the ClinCheck^®^ setup will be faithfully reproduced in vivo; anticipating which movements are most likely to be underexpressed allows clinicians to plan additional auxiliaries, including skeletal anchorage with mini-screws (TADs), from the outset, potentially improving clinical accuracy and reducing biomechanical side effects that could otherwise prolong treatment.

### 4.5. Limitations

This study has some limitations that should be considered when interpreting the results. Its retrospective design does not allow control over treatment-related variables and may introduce selection bias, and the unequal sex distribution may further limit generalizability. Measurements were based on superimposition referenced to the palatal rugae, which captures crown movement only; root displacement and compliance wear time could not be objectively verified. Both hemiarches from the same patient were analyzed as separate units, which may introduce a degree of statistical non-independence, although this is consistent with the bilateral sample size calculation performed. Finally, the study focused on a single aligner system, so findings may not be directly generalizable to other aligner materials or appliance designs. Future prospective studies with larger, more balanced samples, root-level analysis, objective compliance monitoring, evaluation of refinement effects, and long-term follow-up are warranted.

## 5. Conclusions

1. The predictability of sequential distalization in the upper arch has been shown to be unreliable in molars, canines, and incisors. The planned distalization of 2.60 and 2.45 mm for first molars and canines, respectively, resulted in actual distal movements of approximately 1.80 and 1.12 mm, as observed in the CAT. Given that the planned movements were only partially achieved after the initial treatment plan, and due to this lack of predictability, subsequent refinement stages should be anticipated later in treatment to correct the initial objectives.
2. Molar distalization led to distal inclination movement and corono-buccal torque, as well as unplanned intrusion. The use of vertical rectangular attachments was associated with better control over molar crown inclination.

## Figures and Tables

**Table 1:** Predicted, achieved and deviational movements of maxillary first molars.

Molar Movement	Predicted Mean ± SD	Achieved Mean ± SD	Deviational Movement Mean ± SD	*p*
Extrusion/Intrusion (mm)	−0.01 ± 0.40	0.79 ± 0.52	0.80 ± 0.53	0.000 *
Translation B/L (mm)	1.17 ± 0.90	1.14 ± 0.77	−0.04 ± 0.44	0.462
Translation M/D (mm)	2.60 ± 1.42	1.80 ± 1.09	−0.80 ± 0.71	0.000 *
Rotation (°)	6.28 ± 5.21	5.55 ± 4.92	−0.74 ± 2.48	0.008 *
Angulation (°)	2.83 ± 2.89	6.51 ± 3.56	3.69 ± 2.47	0.000 *
Torque (°)	0.57 ± 4.38	4.12 ± 3.88	3.55 ± 2.88	0.000 *

Abbreviations: B/L, buccolingual; M/D, mesiodistal; SD, standard deviation. Positive values indicate distalization, intrusion, or buccal displacement. * *p* < 0.05.

**Table 2:** Predicted, achieved and deviational movements of maxillary canines.

Canine Movement	Predicted Mean ± SD	Achieved Mean ± SD	Deviational Movement Me an± SD	*p*
Extrusion/Intrusion (mm)	0.12 ± 0.74	−0.03 ± 1.45	−0.14 ± 1.32	0.337
Translation B/L (mm)	−0.35 ± 1.18	0.00 ± 1.08	0.36 ± 1.55	0.042 *
Translation M/D (mm)	2.45 ± 1.36	1.12 ± 0.99	−1.33 ± 1.01	0.000 *
Rotation (°)	9.61 ± 12.12	5.14 ± 7.25	−4.47 ± 7.01	0.000 *
Angulation (°)	0.69 ± 5.65	2.80 ± 4.97	2.11 ± 7.11	0.009 *
Torque (°)	3.20 ± 4.82	4.20 ± 4.29	1.01 ± 2.17	0.000 *

Abbreviations: B/L, buccolingual; M/D, mesiodistal; SD, standard deviation. Positive values indicate distalization, intrusion, or buccal displacement. * *p* < 0.05.

**Table 3:** Predicted, achieved and deviational movements of maxillary incisors.

Incisor Movement	Predicted Mean ± SD	Achieved Mean ± SD	Deviational Movement Mean ± SD	*p*
Extrusion/Intrusion (mm)	1.45 ± 1.12	0.03 ± 0.82	−1.42 ± 1.23	0.000 *
Translation B/L (mm)	0.76 ± 2.15	−0.09 ± 1.64	−0.86 ± 1.07	0.000 *
Translation M/D (mm)	−0.13 ± 1.20	−0.07 ± 0.81	0.05 ± 0.98	0.628
Rotation (°)	2.62 ± 13.63	1.63 ± 8.85	−0.99 ± 6.62	0.184
Angulation (°)	1.69 ± 3.81	0.91 ± 2.90	−0.78 ± 1.36	0.000 *
Torque (°)	7.59 ± 8.56	1.76 ± 6.37	−5.83 ± 4.57	0.000 *

Abbreviations: B/L, buccolingual; M/D, mesiodistal; SD, standard deviation. Positive values indicate distalization, intrusion, or buccal displacement. * *p* < 0.05.

**Table 4:** Accuracy of molar, canine, and incisor movements according to planned tooth movement, expressed as the percentage of the planned displacement that was actually achieved.

Movement	Molar Accuracy (%)	Canine Accuracy (%)	Incisor Accuracy (%)
Extrusion/Intrusion	— a	— b	2.1
Translation B/L	97.4	— a	— a
Translation M/D	69.2	45.7	— b
Rotation	88.4	53.5	62.2
Angulation	— c	— c	53.8
Torque	— c	— c	23.2

a Reversed: The achieved movement occurred in the opposite direction to that which was planned. b Not applicable: The planned value was close to zero. c Overexpressed: The achieved value exceeded the planned value.

**Table 5:** Multivariable regression analysis of factors associated with molar movements.

**Maxillary First Molar**	**Extrusion/Intrusion (mm)**		**Translation B/L (mm)**		**Translation M/D (mm)**	
	**B (CI)**	***p***	**B (CI)**	***p***	**B (CI)**	***p***
Optimized Attachment	−0.181 (−0.461, 0.099)	0.200	−0.086 (−0.305, 0.132)	0.431	0.004 (−0.348, 0.339)	0.980
Rectangular Attachment	Reference		Reference		Reference	
ANB	−0.059 (−0.155, 0.037)	0.224	−0.027 (−0.103, 0.049)	0.482	0.020 (−0.102, 0.143)	0.738
Mandibular Plane	0.006 (−0.024, 0.037)	0.688	0.003 (−0.026, 0.020)	0.779	−0.025 (−0.064, 0.013)	0.192
Number of Aligners	0.003 (−0.014, 0.019)	0.763	0.005 (−0.008, 0.018)	0.454	−0.006 (−0.027, 0.013)	0.505
**Maxillary First Molar**	**Rotation (°)**		**Angulation (°)**		**Torque (°)**	
	**B (CI)**	***p***	**B (CI)**	***p***	**B (CI)**	***p***
Optimized Attachment	0.725 (−0.707, 2.220)	0.335	**1.430 (0.031, 2.828)**	0.045 *	−0.737 (−2.242, 0.776)	0.330
Rectangular Attachment	Reference		Reference		Reference	
ANB	−0.033 (−0.528, 0.461)	0.892	0.106 (−0.375, 0.588)	0.659	−0.005 (−0.532, 0.522)	0.985
Mandibular Plane	**−0.212 (−0.372, −0.052)**	0.010 *	−0.130 (−0.279, 0.019)	0.086	−0.137 (−0.300, 0.026)	0.099
Number of Aligners	0.029 (−0.056, 0.114)	0.505	0.017 (−0.066, 0.101)	0.680	0.001 (−0.087 to 0.090)	0.969

Abbreviations: B/L, buccolingual; M/D, mesiodistal; B, regression coefficient; CI, confidence interval; ANB, A point–Nasion–B point angle. Reference category for attachment type: rectangular attachment. Significant coefficients (*p* < 0.05) are shown in bold. * *p* < 0.05.

**Table 6:** Multivariable regression analysis of factors associated with canine movements.

**Maxillary Canine**	**Extrusion/Intrusion (mm)**		**Translation B/L (mm)**		**Translation M/D (mm)**	
	**B (CI)**	***p***	**B (CI)**	***p***	**B (CI)**	***p***
Optimized Attachment	−0.210 (−0.958, 0.537)	0.576	−0.100 (−0.690, 0.489)	0.736	−0.158 (−0.525, 0.208)	0.392
Rectangular Attachment	Reference		Reference		Reference	
ANB	−0.125 (−0.351, 0.099)	0.270	−0.035 (−0.221, 0.142)	0.693	−0.056 (−0.166, 0.053)	0.308
Overbite	0.090 (−0.102, 0.283)	0.353	0.003 (−0.120, 0.127)	0.958	**−0.071 (−0.147, 0.003)**	0.062 *
Overjet	−0.016 (−0.289, 0.256)	0.906	0.051 (−0.157, 0.260)	0.622	−0.095 (−0.224, 0.032)	0.142
**Maxillary Canine**	**Rotation (°)**		**Angulation (°)**		**Torque (°)**	
	**B (CI)**	***p***	**B (CI)**	***p***	**B (CI)**	***p***
Optimized Attachment	1.242 (−0.735, 3.221)	0.214	−1.824 (−4.156, 0.506)	0.123	−0.408 (−1.494, 0.677)	0.455
Rectangular Attachment	Reference		Reference		Reference	
ANB	−0.447 (−1.050, 0.156)	0.144	0.289 (−0.403, 0.982)	0.407	0.077 (−0.239, 0.393)	0.628
Overbite	−0.034 (−0.446, 0.378)	0.870	−0.846 (−1.333, 0.359)	0.001	0.167 (−0.059, 0.394)	0.145
Overjet	−0.281 (−0.978, 0.415)	0.422	−1.202 (−2.024, 0.380)	0.005	−0.174 (−0.547, 0.198)	0.353

Abbreviations: B/L, buccolingual; M/D, mesiodistal; B, regression coefficient; CI, confidence interval; ANB, A point–Nasion–B point angle. Reference category for attachment type: rectangular attachment. Significant coefficients (*p* < 0.05) are shown in bold. The duplicate blank row present in the original table has been removed. * *p* < 0.05.

**Table 7:** Multivariable regression analysis of factors associated with incisor movements.

**Maxillary Incisor**	**Extrusion/Intrusion (mm)**		**Translation B/L (mm)**		**Translation M/D (mm)**	
	**B (CI)**	***p***	**B (CI)**	***p***	**B (CI)**	***p***
Without Attachment	−0.005 (−0.381, 0.370)	0.976	0.047 (−0.421, 0.516)	0.840	−0.023 (−0.399, 0.351)	0.900
With Attachment	Reference		Reference		Reference	
Without Power Ridge	0.062 (−0.297, 0.421)	0.732	−0.412 (−0.858, 0.034)	0.070 *	0.108 (−0.259, 0.475)	0.559
With Power Ridge	Reference		Reference		Reference	
ANB	−0.0435 (−0.143, 0.056)	0.386	0.000 (−0.122, 0.120)	0.988	−0.029 (−0.128, 0.069)	0.554
Overbite	**0.138 (0.040, 0.236)**	0.014 *	−0.017 (−0.105, 0.070)	0.693	**−0.088 (−0.158, −0.018)**	0.014 *
Overjet	**−0.189 (−0.310, 0.068)**	0.003 *	0.048 (−0.126, 0.223)	0.582	−0.038 (−0.156, 0.080)	0.521
**Maxillary Incisor**	**Rotation (°)**		**Angulation (°)**		**Torque (°)**	
	**B (CI)**	***p***	**B (CI)**	***p***	**B (CI)**	***p***
Without Attachment	−0.579 (−2.7436, 1.576)	0.594	−0.060 (−0.597, 0.476)	0.818	**−2.422 (−4.232, −0.612)**	0.009 *
With Attachment	Reference		Reference		Reference	
Without Power Ridge	0.544 (−1.516, 2.604)	0.600	0.119 (−0.383, 0.622)	0.638	**−3.788 (−5.636, −1.940)**	0.000 *
With Power Ridge	Reference		Reference		Reference	
ANB	0.330 (−0.236, 0.898)	0.249	−0.042 (−0.181, 0.096)	0.544	−0.173 (−0.650, 0.303)	0.470
Overbite	−0.020 (−0.424, 0.382)	0.918	−0.018 (−0.116, 0.079)	0.708	−0.192 (−0.562, 0.176)	0.302
Overjet	0.073 (−0.569, 0.717)	0.820	0.015 (−0.144, 0.175)	0.849	−0.228 (−0.887, 0.430)	0.492

Abbreviations: B/L, buccolingual; M/D, mesiodistal; B, regression coefficient; CI, confidence interval; ANB, A point–Nasion–B point angle. Reference category: with attachment/with Power Ridge. Significant coefficients (*p* < 0.05) are shown in bold. * *p* < 0.05.

## Data Availability

The data supporting the findings of this study are available from the corresponding author upon reasonable request.
